# Rare Case of a Young Female With Co-existent Hydatidiform Mole and Pulmonary Metastases: An Underrecognized Entity

**DOI:** 10.7759/cureus.20245

**Published:** 2021-12-07

**Authors:** Ravikanth Reddy

**Affiliations:** 1 Radiodiagnosis, St. John's Hospital, Bengaluru, IND

**Keywords:** beta-human chorionic gonadotrophin levels, gestational trophoblastic disease, combination chemotherapy, pulmonary metastases, hydatidiform mole

## Abstract

Gestational trophoblastic disease (GTD) comprises placental-site hydatidiform moles, invasive moles, or choriocarcinoma which are of unknown etiology and characterized by abnormal proliferation of gestational trophoblastic tissue. Furthermore, malignant GTD is also characterized by hematogenous spread to distant metastatic sites. Nevertheless, early diagnosis of gestational trophoblastic disease is important to ensure timely and successful management of the clinical condition and for the preservation of fertility. We report the unusual case of a complete hydatidiform mole to pulmonary metastases in a 27-year-old woman with elevated beta-human chorionic gonadotropin (β-hCG) levels. The placental histopathology showed a complete hydatidiform mole with absent fetal parts. Beta-human chorionic gonadotrophin (β-hCG) levels were found elevated at 893 mIU/mL. The case was discussed at the multidisciplinary tumour board and surgical resection with four cycles of combination chemotherapy was recommended, following which β-hCG normalization was achieved. This case report highlights the importance of clinical vigilance even in low-risk patients. Unexpected findings on ultrasound should involve multidisciplinary input from radiologists and surgical oncologists. A high index of suspicion for gestational trophoblastic disease and imaging follow-up for metastases is imperative.

## Introduction

The spectrum of clinical entities ranging from benign forms such as hydatidiform mole to malignant forms such as choriocarcinoma comprises gestational trophoblastic disease (GTD). Nevertheless, the greatest challenge in the management of gestational trophoblastic disease is the decision to initiate chemotherapy after the evacuation of the molar pregnancy to prevent possible progression to gestational trophoblastic neoplasia. Chemotherapeutic regimens with methotrexate have been recommended for the management of molar pregnancies, such as partial and complete hydatidiform moles [1]. Post-molar follow-up involves the serial detection of the tumor-specific quantitative marker beta-human chorionic gonadotrophin (β-hCG) [2]. Chest radiographs and computed tomography (CT) of the chest are recommended radiological investigations, when pulmonary metastatic disease is suspected. The spectrum of entities in gestational trophoblastic disease occurs mostly in women of reproductive age and is extremely rarely encountered in post-menopausal women [3]. Following the diagnosis of hydatidiform mole, evacuation of the molar pregnancy should be performed at the earliest to minimize the risk of hematogenous metastases. Pulmonary deportation of gestational trophoblastic disease has been described in partial mole, invasive mole, or choriocarcinoma. However, complete hydatidiform mole is a benign form of gestational trophoblastic disease with an extremely rare incidence of pulmonary metastases. We report the unusual case of a complete hydatidiform mole to pulmonary metastases in a 27-year-old woman with elevated beta-human chorionic gonadotropin (β-hCG) levels.

## Case presentation

**Figure 1:**
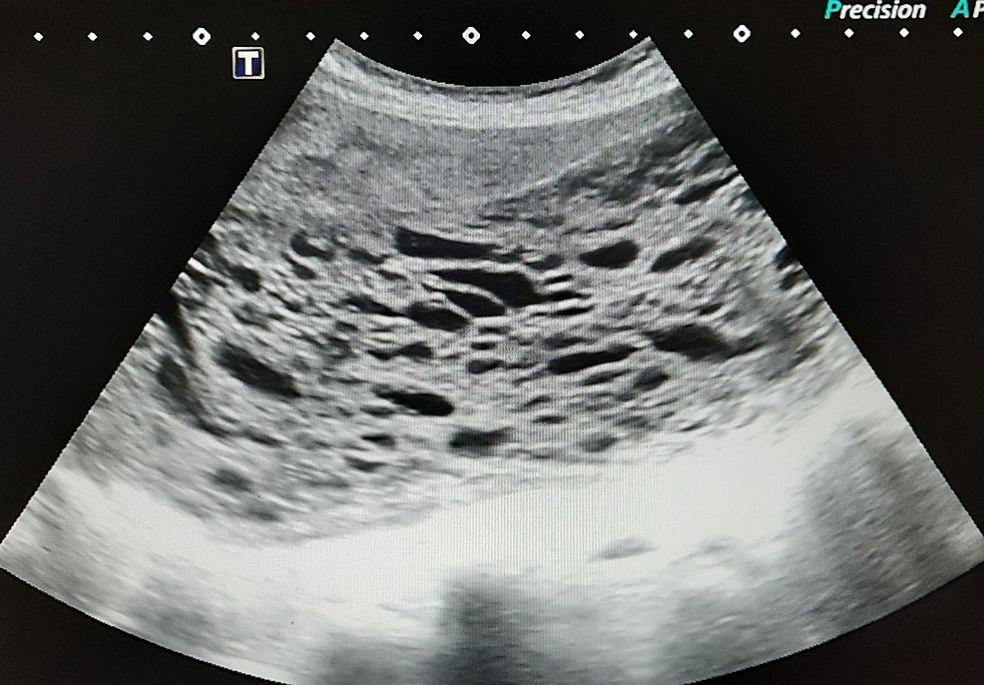
Transverse ultrasonography image demonstrating a heterogeneous intrauterine mass containing multiple cystic spaces. Note the absent fetal parts with an associated snow-storm appearance consistent with features of complete molar pregnancy.

**Figure 2:**
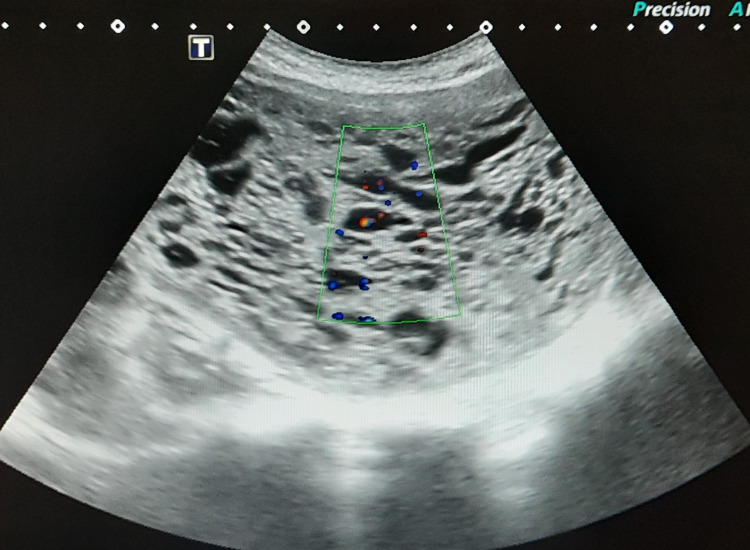
Color Doppler ultrasonography image demonstrating no vascularity within intrauterine mass with cystic spaces.

**Figure 3:**
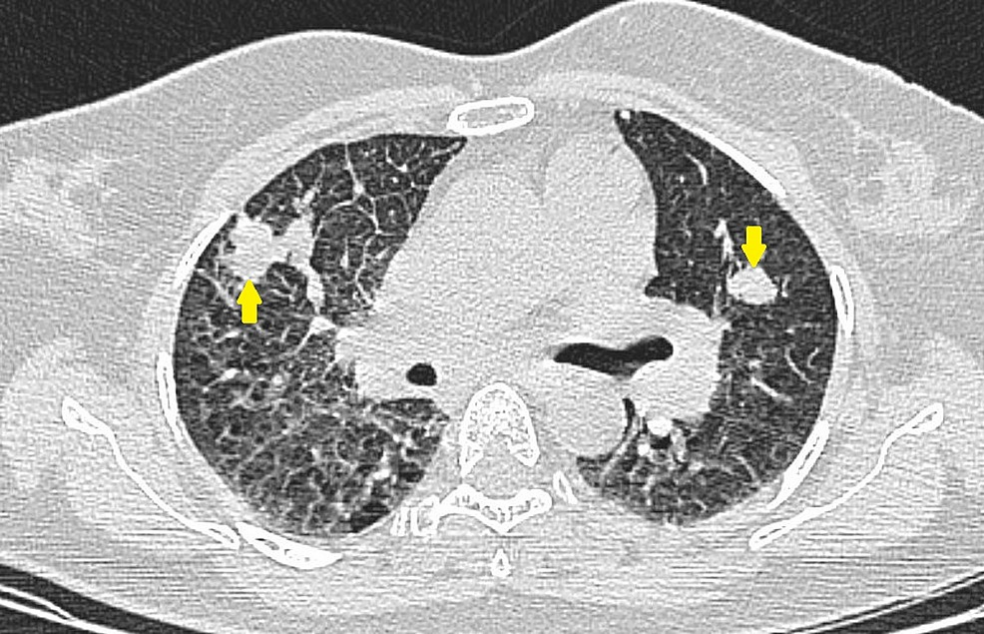
CECT of chest demonstrating metastatic lesions (arrows) in bilateral lungs with nodular and irregular interlobular septal thickening consistent with features of lymphangitis carcinomatosa. CECT: contrast-enhanced computed tomography.

**Figure 4:**
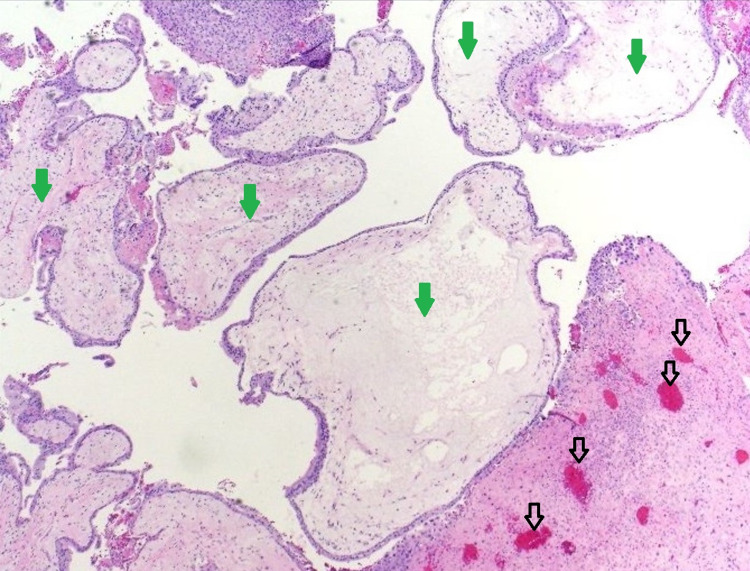
Histopathology image of the evacuated specimen demonstrating trophoblastic proliferation (hollow black arrows) and hydropic degeneration of villi (solid green arrows) consistent with features of molar pregnancy (H and E, ×400).

A 27-year-old nulliparous woman presented with progressive lower abdominal pain since 10 days and occasional hemoptysis since two months. She was referred to the department of obstetrics for further evaluation after her urine pregnancy test showed positivity and her last menstrual period was 19+4 weeks prior to presentation. The patient was hemodynamically stable (BP 110/70 mmHg, HR 74 bpm), and her electrocardiogram was unremarkable. The abdomen was distended and a huge mass was palpable above the level of the umbilicus. Pelvic examination with a speculum showed no blood in the vagina and a closed cervical os. However, the subsequent ultrasound revealed a distended endometrial cavity containing a heterogeneous intrauterine mass with multiple cystic spaces demonstrating snow storm appearance with absent fetal parts suggestive of complete molar pregnancy (Figures 1, 2). Beta-human chorionic gonadotrophin (β-hCG) levels were found elevated at 893 mIU/mL. Routine chest radiograph revealed evidence of suspicious lesions in bilateral upper lung zones which were further investigated on contrast-enhanced computed tomography (CECT) of the chest thus confirming pulmonary metastases (Figure 3). The patient underwent suction curettage and a large amount of trophoblastic tissue with clots was evacuated. On gross examination, the molar specimen consisted of clots admixed with grape-like vesicles. Histopathology examination of the evacuated specimen confirmed the diagnosis of molar pregnancy (Figure 4). Following surgical resection, the patient was rescheduled for combination chemotherapy with methotrexate. The patient was discharged from the hospital, and after an uneventful discharge, she underwent a total of four cycles of etoposide, methotrexate, actinomycin D, cyclophosphamide, vincristine (EMA/CO) adjuvant consolidation chemotherapy regimen on a regular basis in an inpatient hospital unit. No other evidence of metastatic disease or recurrence has been noted over 24 months of post-operative follow-up.

## Discussion

Hydatidiform mole is a product of anomalous conceptions, with a prevalence of about one in 500-1,000 pregnancies [4]. All cases of molar pregnancy are categorized as complete or partial hydatidiform moles. In complete molar pregnancy diffuse swelling of chorionic villi and disseminated thromboplastic hyperplasia without embryo or fetal tissues is characteristic. These cases commonly have a diploid karyotype. Partial molar pregnancy demonstrates fetal parts and consistently bears an association with gestational trophoblastic neoplasia, anemia, and congenital anomalies, such as cleft lip and syndactyly [5]. Patients who are diagnosed with molar pregnancy must be evaluated for possible complications, such as overactive thyroid, anemia, and toxemia of pregnancy [6]. Patients are monitored to prevent the recurrence of benign moles and the development of malignant neoplasia, which can metastasize to the brain, liver, or lungs. Recurring moles are treated with low-level chemotherapy using methotrexate [7]. Ultrasound is the modality of choice for diagnosing localized gestational trophoblastic disease. Molar pregnancy is characteristically observed as an enlarged uterus containing a hyperechoic, solid, heterogenous mass, with multiple cystic spaces representing dilated, hydropic villi. Bilateral ovarian theca lutein cysts are present in 30-50% of cases [8]. The most peculiar biologic characteristic of trophoblasts is their ability to erode maternal tissue. Even in normal pregnancies, trophoblasts have the potential for invasion and metastasis. Notwithstanding the aggressive behavior and high metastatic rate of hydatidiform moles, they are also highly sensitive to chemotherapy, which is considered the first-line treatment [9]. The American College of Obstetricians and Gynecologists has recommended that after the evacuation of a mole, serum β-hCG levels should be monitored every one to two weeks in all patients while the levels are elevated and then at monthly intervals for an additional six months once the levels become undetectable [10].

Invasive mole due to its aggressive behavior of local invasion of myometrium and adnexa is considered a malignant entity along the spectrum of tumor progression to gestational trophoblastic neoplasia. The metastatic rate of an invasive mole is high with 30% of the cases demonstrating metastasis at the time of initial presentation with the most common sites of metastases being the lung (30%), followed by the vagina (30%), the liver (10%), and less commonly to the breast, bones, and brain [11]. Nevertheless, imaging of the lungs is recommended for all patients with gestational trophoblastic disease. Also, there is added risk of developing abdominal and central nervous system metastases in patients of gestational trophoblastic disease with pulmonary metastases. The pattern of metastases reiterates the role of abdominal CT and brain MRI in the staging process of gestational trophoblastic disease. Choriocarcinoma is characterized by hypervascularity and rapid proliferation of the tumor cells leading to spontaneous hemorrhage from the metastatic sites which may result in varying imaging appearances. Furthermore, metastatic involvement of the pleura and cavitary metastases may add to the risk of developing pneumothorax [12].

Fluorodeoxyglucose positron emission tomography (FDG-PET) with a reported sensitivity of 87% for the detection of pulmonary metastases is considered to be a sensitive tool for detecting distant metastases [13]. Combination chemotherapy regimens are recommended for the treatment of gestational trophoblastic disease and also have an important role in the management of metastatic gestational trophoblastic neoplasia with satisfactory results obtained during the initial treatment period. However, currently surgical resection of pulmonary metastatic lesions is reserved for relapsed lesions or new-onset lesions of metastatic pulmonary disease with co-existent gestational trophoblastic disease [14].

## Conclusions

In conclusion, this case report deserves special mention as it highlights the importance of timely management in patients with hydatidiform mole which might require chemotherapy and/or surgical resection when there is the concomitant occurrence of pulmonary metastases which is truly an underrecognized entity. A meticulous pulmonary workup including chest radiographs, computed tomography, and FDG-PET may be required either as the initial investigation or during the follow-up period for early detection of pulmonary metastases which although rare are very much a possibility and are frequently encountered with almost any entity encountered alongside the clinical spectrum of gestational trophoblastic disease. This case report also highlights the need for clinical correlation, precise diagnosis, and appropriate management, with subsequent follow-up in the setting of gestational trophoblastic disease due to the possibility of rapid progression and increased mortality from pulmonary metastases despite adequate response to chemotherapy.
